# A Therapeutic and Diagnostic Dilemma: Granular Cell Tumor of the Breast

**DOI:** 10.1155/2011/972168

**Published:** 2011-04-06

**Authors:** Ahmet Pergel, Ahmet Fikret Yucel, A. Serdar Karaca, Ibrahim Aydin, Dursun Ali Sahin, Nilgun Demirbag

**Affiliations:** ^1^Department of Surgery, Rize University School of Medicine, 53100 Rize, Turkey; ^2^Clinics of Surgery, Bartin State Hospital, 74000 Bartin, Turkey; ^3^Department of Pathology, Avrasya Hospital, 34010 Istanbul, Turkey

## Abstract

Six to eight percent of granular cell tumors are seen in the breast. Although mostly benign, they rarely have malignant features clinically and radiologically reminding of breast cancer. This may lead to a potential misdiagnosis of breast carcinoma and overtreatment of patients. The final diagnosis is made by immunohistochemical examination. We performed excisional biopsy on a patient who was diagnosed to have a breast mass. The histopathological examination of the mass revealed granular cell tumor.

## 1. Introduction

Granular cell tumor (GCT) of the breast is usually benign [1, 2]. It rarely shows malignant features [3]. Initially it was thought to originate from skeletal muscle cells but due to S-100 protein positivity and the similarity of the tumor cells to Schwann cells, researchers concluded that the tumor originated from the Schwann cells between the lobular breast tissue [2, 4]. Herein, we present a patient who underwent excisional biopsy of a breast mass which was diagnosed as granular cell tumor as a result of histopathological examination.

## 2. Case Presentation

**Figure 1:**
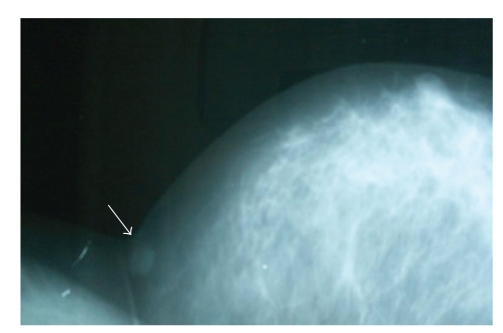
Mass in the axillary tail of the left breast (mammographic image).

**Figure 2:**
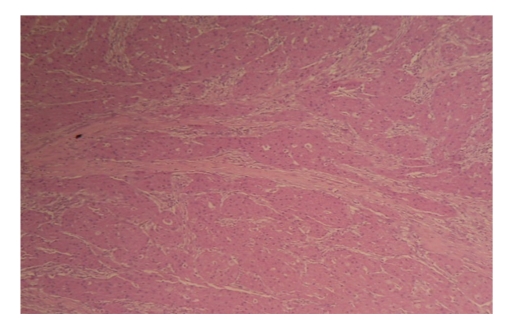
Nests of polygonal cells with eosinophilic cytoplasm divided by fibrous septa (H&E, ×40).

**Figure 3:**
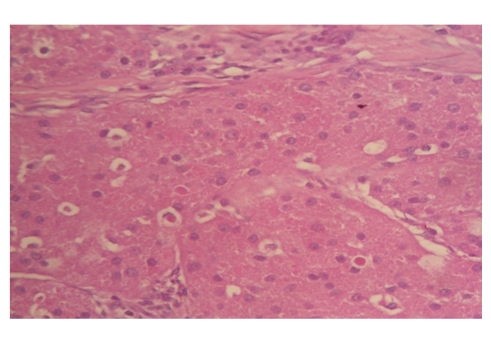
Eosinophilic granular cells with vesicular nuclei, prominent nucleoli, and in addition intracytoplasmic eosinophilic particles surrounded by a clear zone (H&E, ×100).

**Figure 4:**
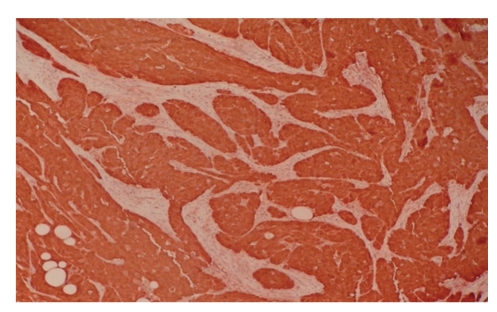
Tumor cells showing diffuse strong immunostaining for S-100 protein (×40).

**Figure 5:**
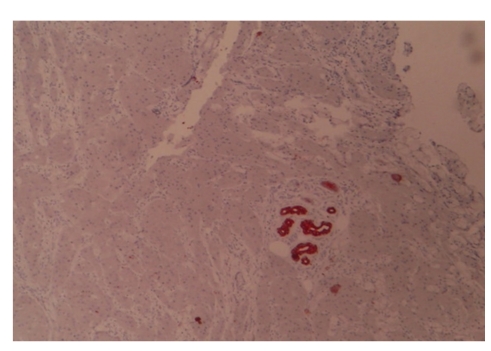
The lack of GCDFP-15 immunostaining in tumor cells (×40).

Thirty-five-year-old female presented to our clinic with complaint of a mass in her left breast. Physical examination revealed a 15 × 15 mm painless mass in upper outer quadrant of the left breast under the subcutaneous tissue. Examination of the other breast and axilla was normal. Ultrasonography showed a 15 mM diameter solid mass which had mild acoustic shadowing in the posterior aspect. Mammography revealed a smooth bordered opacity with a 1 cm diameter in the axillary tail of the left breast (Figure 1). With these findings, the mass was considered as benign and the lesion was removed with some healthy tissue around it. The histological examination revealed polygonal cells with eosinophilic granular cytoplasm, and fibrous septae between the clusters (Figure 2), cells with vesicular nuclei with prominent nucleoles and eosinophilic granular cytoplasms and eosinophilic intracytoplasmic particles surrounded by a clear halo (Figure 3). In immunohistochemical examination, S-100 protein (Figure 4), CEA, and vimentin were (+), and cytokeratin was (−) (Figure 5), and these findings led us to the diagnosis of granular cell tumor.

## 3. Discussion

 GCT often seen in the premenopausal period. Although estrogen and progesterone have been thought to play a role in GCT pathogenesis, in most cases, hormone receptors are negative. It presents as a painless breast mass in women. The most frequent location is the upper middle and medial quadrant [2]. Clinically it may mimic breast cancer causing nipple and/or skin retraction [5]. It may also mimic breast cancer in mammography or ultrasonography although it mostly presents as a well-circumscribed mass [6]. Diagnostic imaging presentation of GCT of the breast is changeable. These lesions have been defined as ranging from a round well-circumscribed mass to an indistinct or spiculated lesion on mammography. Microcalcifications are not normally a feature of GCTs. On ultrasound, GCTs can present as solid, poorly marginated lesions with marked posterior shadowing or as more benign-appearing well-circumscribed solid masses [7, 8]. In our case, the mass was in the upper outer quadrant and was superficial and mobile. There was no skin retraction. Breast ultrasound and mammography revealed a well-circumscribed mass of 15 mM diameter without spicular extension and microcalcification. 

Fine-needle aspiration biopsy and frozen section methods are inadequate for definitive diagnosis of GCT but are helpful in differentiating the lesion from apocrine carcinoma, histiocytic variant of invasive lobular carcinoma and metastatic carcinomas [9]. In our case, as the mass was superficial and no signs of malignancy were present, we preferred excisional biopsy. 

The definitive diagnosis of GCT is only possible with immunohistochemical examination. S-100 positivity and cytokeratin negativity lead to the diagnosis of GCT [1]. CD68, CEA, and vimentin were reported to be positive in some cases in the literature [9]. In our case, S-100, CEA, and vimentin positivity and cytokeratin negativity led to the diagnosis of GCT. 

Wide excision is sufficient for the treatment of GCT. Subtotal excision may lead to local recurrences [6]. In our case, the tumor was removed with surrounding normal tissue which obviated the need for a second operation. 

## 4. Conclusion

 GCT of the breast is a usually benign disease of the breast which may mimic breast cancer both clinically and radiologically. The definitive diagnosis is by immunohistochemical examination. It should be kept in mind while dealing with breast masses to prevent overtreatment. 

##  Conflict of Interests

The authors declare that they have no competing interests.
